# Hodgkin Lymphoma Classification—From Historical Concepts to Current Refinements

**DOI:** 10.3390/cancers17172929

**Published:** 2025-09-07

**Authors:** Antonino Carbone, Mohamed N. Alibrahim

**Affiliations:** 1 Centro di Riferimento Oncologico, Istituto di Ricovero e Cura a Carattere Scientifico, National Cancer Institute, 33081 Aviano, Italy; 2 Faculty of Medicine, Zagazig University, Zagazig 44519, Egypt; 20512020101186@medicine.zu.edu.eg

**Keywords:** Hodgkin lymphoma, classic Hodgkin lymphoma, NLPHL, classification, WHO classification 5th edition, International Consensus Classification

## Abstract

The current classification of Hodgkin lymphoma (HL) is the result of an integrated approach that combines the evaluation of morphological patterns, immunophenotypic characteristics, molecular features, and clinical presentation. Evolving from its origins based solely on histological observation to the latest updates in the WHO 5th Edition, this system has become an essential tool for accurate diagnosis and personalized therapeutic strategies. Each subtype of classic HL (cHL)—nodular sclerosis, mixed cellularity, lymphocyte-rich, and lymphocyte-depleted—exhibits distinctive pathological and clinical features, now better understood through multidisciplinary studies and international collaborations. HL also includes nodular lymphocyte-predominant HL (NLPHL), a distinct entity with unique morphologic, immunophenotypic, and clinical features. A hallmark morphological feature of cHL is the presence of Hodgkin and Reed-Sternberg (HRS) cells, large and often multinucleated cells derived from B lymphocytes that have lost their typical B-cell phenotype. Identifying these cells is critical for diagnosis and for differentiating HL from other hematologic malignancies. HL is characterized by the rarity of malignant cells, a high curability rate, and a rich immune cell microenvironment that is both shaped and exploited by the tumor. Understanding these core aspects paves the way for exploring the role of immunologic and genetic biomarkers in refining classification, enhancing diagnosis, improving prognostic assessment, and guiding therapy for patients with cHL.

## Simple Summary

Hodgkin Lymphoma (HL) represents a distinct type of B-cell—derived lymphoid malignancy characterized by several unique clinical and pathological features. Among these, a hallmark of HL is its age distribution which shows two peaks of incidence, its generally favorable prognosis and high curability. Morphological evaluation remains the cornerstone of HL diagnosis, although the evolving landscape of HL classification reflects an integrated understanding of morphological patterns, immunophenotypic profiles, molecular features, and clinical behavior. From its historical roots to the most recent refinements in the WHO 5th Edition and in International Clinical Consensus (ICC) classification, the classification system is used as a scientific basis for a precise diagnosis and patient care.

## 1. Introduction

Hodgkin lymphoma (HL) is a distinctive malignancy of B-cell origin, marked by characteristic clinical and pathological hallmarks [1,2]. One of its defining epidemiologic patterns is a bimodal age distribution, with a primary incidence peak in young adulthood and a secondary, smaller peak in older age [3]. Clinically, HL is notable for its excellent prognosis, with most patients achieving durable remission when treated with contemporary therapeutic regimens—an outcome that distinguishes it from many other forms of lymphoma [4,5].

Classic Hodgkin lymphoma (cHL) accounts for roughly 90–95% of HL cases and is categorized into four histologic subtypes: nodular sclerosis (NScHL), mixed cellularity (MCcHL), lymphocyte-rich (LRcHL), and lymphocyte-depleted (LDcHL) [6]. The remaining ~5% of cases are nodular lymphocyte-predominant Hodgkin lymphoma (NLPHL), a distinct clinicopathological entity characterized by ‘popcorn’ or LP cells with a B-cell phenotype (CD20+, CD45+), typically presenting with localized peripheral lymphadenopathy, an indolent course, and a tendency for late relapses [7].

cHL forms differ in clinical behavior and immune interactions. NScHL is the most prevalent subtype, especially among adolescents and young adults, and frequently presents with mediastinal lymph node involvement [4].

Epstein–Barr virus (EBV) infection is an established etiologic factor for certain cHL subtypes, particularly MCcHL and LDcHL. EBV-associated HL is more common in children, older adults, and immunocompromised groups, including those with HIV/AIDS or solid organ transplants [8,9,10]. In individuals living with HIV, HL is almost always EBV-positive, and the introduction of antiretroviral therapy has shifted the predominant subtype toward NScHL [9,10,11,12].

The tumor microenvironment (TME) plays a central role in the pathogenesis and progression of cHL. Unlike most malignancies where neoplastic cells constitute the bulk of the tumor mass, HL lesions contain only a small fraction of malignant Hodgkin and Reed–Sternberg (HRS) cells [13,14,15]. The majority of the tumor is composed of a diverse array of immune and stromal cells—T cells, B cells, eosinophils, macrophages, and fibroblasts. HRS cells actively manipulate this microenvironment to evade immune surveillance and promote tumor growth [14,16].

Understanding these core features provides a framework for advancing the use of immunologic and genetic biomarkers to enhance diagnostic precision, refine prognostic evaluation, and optimize therapeutic approaches in patients with cHL.

## 2. Evolution of Classification Systems

The classification of HL has undergone substantial refinement over time, shaped by advancements in morphological assessment, immunophenotyping, and molecular diagnostics. This paper examines the historical and modern evolution of HL classification, emphasizing the updates introduced in the World Health Organization WHO 5th Edition [6] and contrasting them with the International Consensus Classification (ICC) [17,18].

### 2.1. Historical Classification of Hodgkin Lymphoma

Earlier classification systems preceding the Rye scheme already included what is now recognized as Hodgkin lymphoma. The Jackson and Parker classification of 1944 divided HL into paragranuloma, granuloma, and sarcoma, although it was later considered clinically limited since most patients clustered in the granuloma subtype [19]. The Rappaport classification of 1956 introduced a cytological and architectural approach, distinguishing nodular versus diffuse patterns across lymphomas, which also encompassed cases of Hodgkin’s disease [20]. These frameworks laid the foundation for the Lukes–Butler (1964) [20] and Rye (1965) [21] classifications, and ultimately for the REAL and WHO schemes that formally distinguished cHL from NLPHL. Terminology has likewise evolved from “Hodgkin’s disease”to “Hodgkin lymphoma, reflecting its neoplastic nature, and later into the distinct cHL vs NLPHL nomenclature adopted by WHO.

The journey of modern HL classification generally considered to begin with the Rye scheme [22], which divided the disease into four histological subtypes based solely on morphology: lymphocyte predominance, nodular sclerosis, mixed cellularity, and lymphocyte depletion. Although seminal, the Rye system lacked immunophenotypic and molecular insights, limiting its clinical utility.

This was followed by the introduction of the Revised European-American Lymphoma (REAL) classification [23], which brought a more biologically relevant framework by incorporating immunological and genetic features. The REAL system was further refined in the successive WHO classifications of 2001, 2008, 2017, and the 2022 5th edition (WHO-HAEM5), which formalized the division of HL into classic HL and NLPHL [6,24,25], which formalized the division of HL into: classic HL accounting for ~95% of HL cases, NLPHL—a biologically distinct entity with B-cell characteristics.

Within cHL, four subtypes were delineated based on morphological and immunophenotypic traits: NScHL, MCcHL, LRcHL, LDcHL.

While these categories remained stable for two decades, mounting evidence from molecular pathology and clinical data warranted reevaluation—particularly concerning LRcHL and NLPHL.

**Figure 1:**
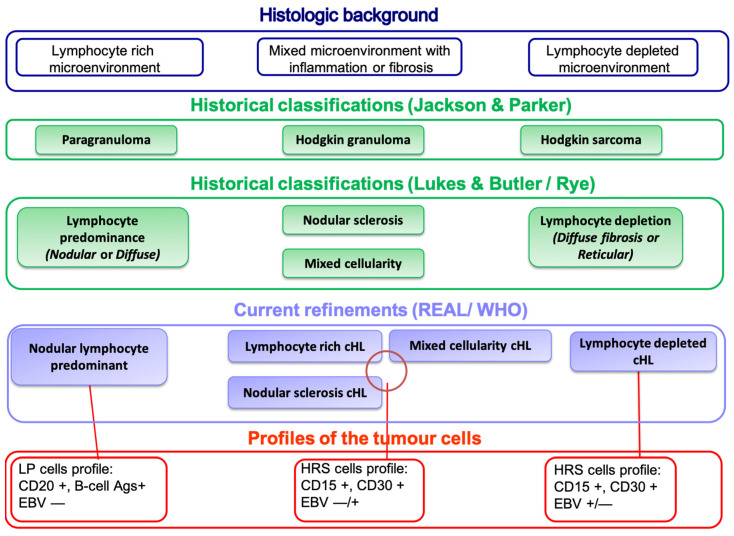
Timeline of Hodgkin lymphoma (HL) classification systems, illustrating the transition from morphology-based subtyping to integrated phenotypic and virologic classification models as proposed by REAL and WHO frameworks. Classic HL subtypes and nodular lymphocyte-predominant HL (NLPHL) are distinguished based on histopathology and biomarker expression.

In summary, the evolution of cHL classification reflects the growing understanding of its molecular and immunologic profile. Early histological systems were gradually replaced by multiparameter approaches, such as the REAL and the WHO classifications. The latter distinguishes cHL—which includes NS, MC, LR, and LD subtypes—from NLPHL, the latter being rarely observed in immunocompromised hosts (Figure 1).

### 2.2. Current WHO 5th Edition Classification of HL

The classification of HL has evolved significantly over the decades, reflecting improvements in morphologic, immunophenotypic, and molecular understanding. The WHO 5th Edition [17] (2022/2023) classification continues to build on this progress, distinguishing HL into two major forms: cHL and NLPHL, with each exhibiting distinct histological and biological profiles.

#### 2.2.1. Recognized Subtypes of HL

Classic Hodgkin Lymphoma

cHL comprises the vast majority of HL cases (~90–95%) and is characterized by the presence of mononuclear Hodgkin and HRS cells in a reactive inflammatory background [4]. cHL is further subclassified into four subtypes: (1) NScHL that is the most common form, particularly affecting adolescents and young adults. Histologically, it features lacunar HRS cells and prominent bands of collagen fibrosis. Immunophenotypically, these cells express CD30 and CD15 but lack the CD45 and B-cell markers, such as CD20 [26]; (2) MCcHL that is the more prevalent in older adults and immunocompromised patients, especially those with HIV, this variant shows abundant classic HRS cells in a heterogeneous background of eosinophils, histiocytes, plasma cells, and neutrophils. It is strongly associated withEBV positivity [27,28,29,30]; (3) LRcHL, a rare subtype that display classic HRS cells within nodules rich in small reactive lymphocytes. These cases may be confused with NLPHL but differ in that they express CD30 and CD15 and often lack CD20. The distinction requires careful morphologic and immunophenotypic analysis [31]; (4) LDcHL that is the least common variant and tends to occur in elderly and HIV-positive patients. It shows diffuse fibrosis, pleomorphic HRS cells, and a paucity of reactive lymphocytes. This aggressive variant has a worse prognosis and is frequently EBV-associated [32,33].

Nodular Lymphocyte-Predominant Hodgkin Lymphoma (NLPHL)

Originally classified under HL, NLPHL is now increasingly recognized as a separate disease entity. The WHO 5th Edition retains its historical label, whereas the ICC redefines it as Nodular Lymphocyte-Predominant B-cell lymphoma (NLPBCL) due to its clinical, morphological, and immunogenetic distinction from cHL [17,18].

The diagnostic features of NLPHL/NLPBCL are the following: (1) tumour cells (so-called “popcorn” or LP cells) exhibit strong expression of CD45 and B-cell markers (CD20, CD79a, BCL6, OCT2, BOB1 [34,35,36]; (2) unlike HRS cells of cHL, LP cells are typically negative for CD15 and CD30 [34,36]; (3) LP cells reside within nodules surrounded by a network of follicular dendritic cells and rosetting T-cells [37]; (4) EBV is almost never associated with NLPHL, unlike certain cHL subtypes (especially MCcHL and LDcHL) [37].

Recent studies emphasize the clinicopathologic spectrum of NLPHL, which includes: (1) incipient forms in which LP cells confined to follicular structures [37,38]; (2) typical NLPHL which is characterized by nodular architecture with strong germinal center B-cell features [39]; (3) transformed forms that resemble T-cell/histiocyte-rich large B-cell lymphoma (THCRLBCL) or even DLBCL, reflecting a continuum of disease progression [40,41]. The Fan system for NLPHL, which identifies six histopathogical grades (A, B, C, D, E and F), could distinguish typical NLPHL (which encompasses grades A, B and C), from the more clinically aggressive tumors (which encompass D, E and F) [40,41].

#### 2.2.2. Diagnostic Criteria (Morphologic and Immunophenotypic) of Classic HL and NLPHL

Accurate diagnosis of HL subtypes requires an integrated approach that combines morphology—identifying HRS or LP cells within characteristic microenvironments [42] and immunohistochemistry using panels that include CD30, CD15, CD20, CD45, BCL6, and related markers. For example, a CD30^+^/CD15^+^ phenotype with loss of B-cell markers supports cHL.” [42].

The WHO 5th Edition has reinforced the distinction between cHL and NLPHL based on robust diagnostic criteria. While cHL is defined by classic HRS cell features within a reactive background, NLPHL exhibits a germinal center B-cell origin characterized by a distinct immunophenotype and clinical behavior [6]. The ICC further advances this distinction by categorizing NLPHL within the spectrum of follicle center B-cell-derived lymphoid neoplasms, reflecting a biologically grounded reclassification [17,18].

These evolving frameworks enhance diagnostic precision, which is essential for guiding targeted therapies and improving patient outcomes.

#### 2.2.3. Key Updates in the WHO 5th Edition (2022/2023)

The WHO 2022/2023 classification refines HL subtyping by recognizing LRcHL as a distinct cHL subtype that, while it may mimic the nodular, lymphocyte-rich architecture of NLPHL, is defined by cHL markers such as CD30 and CD15, highlighting blurred diagnostic boundaries. Greater emphasis is now placed on immunophenotypic profiles: NLPHL shows a B-cell phenotype (CD20, BCL6, CD40) and lacks CD30/CD15, whereas cHL subtypes often co-express CD30 and CD15 despite partial B-cell program retention [43].

Molecular evidence further supports NLPHL as a distinct B-cell lymphoma, given its germinal center origin, ongoing Ig gene mutations, BCL6 translocations, and gene expression patterns more closely resembling THCRLBCL than cHL [40,44]. Moreover, some NLPHL cases have been observed to evolve into THCRLBCL-like proliferations, emphasizing their biological continuum with other large B-cell lymphomas rather than cHL [44].

## 3. Contributions of the International Consensus Classification (ICC)

The ICC [17,18], developed concurrently with the WHO 5th Edition, complements it by reinforcing certain changes and offering alternative perspectives: (1) the ICC concurs with WHO on recognizing NLPHL as a distinct B-cell neoplasm of follicular center origin; (2) It places a higher emphasis on reporting variant growth patterns and transition zones within tumours, which may indicate transformation risk; the ICC advocates for more granular pathology reporting, including the size and extent of diffuse areas in NLPHL, to guide prognosis and treatment planning.

Although there is general harmony between the WHO and the ICC (Table 1), some terminological and emphasis differences remain, reflecting the varied priorities in diagnostic versus clinical practice applications.

**Figure 2:**
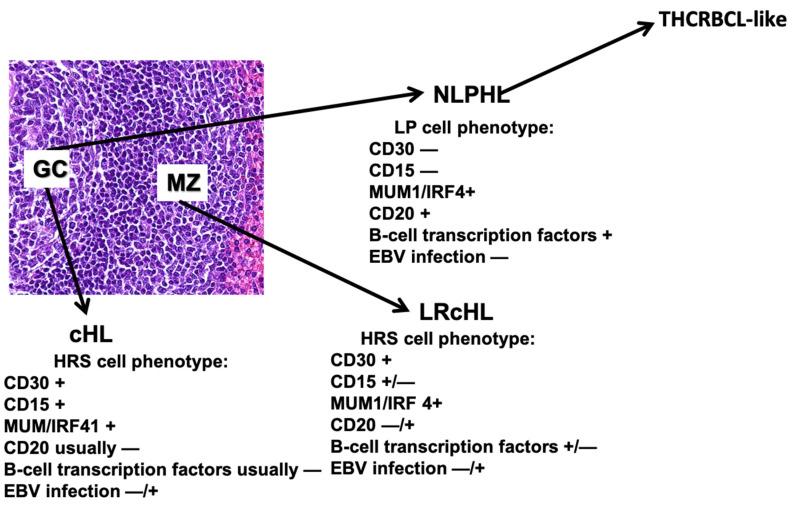
Transitional and Overlapping Features Among Hodgkin Lymphoma Subtypes Nodular lymphocyte-predominant Hodgkin lymphoma (NLPHL) can progress into a diffuse neoplasm enriched with T cells, morphologically and immunophenotypically resembling T-cell/histiocyte-rich large B-cell lymphoma (THCRLBCL). Lymphocyte-rich classic Hodgkin lymphoma (LRcHL) exhibits characteristics that lie between NLPHL and other classic Hodgkin lymphoma (cHL) forms. Notably, LRcHL tumour cells show partial retention of B-cell lineage features while simultaneously expressing cHL-associated markers, including CD30 and CD15, as well as transcription factors indicative of the B-cell program. Abbreviations: cHL, classic Hodgkin lymphoma; EBV, Epstein–Barr virus; GC, germinal center; HRS, Hodgkin and Reed–Sternberg; IRF4, interferon regulatory factor 4; LD, lymphocyte-depleted; LP, lymphocyte-predominant; LRcHL, lymphocyte-rich classic Hodgkin lymphoma; MZ, mantle zone; NLPHL, nodular lymphocyte-predominant Hodgkin lymphoma; PAX5, paired box gene 5; MC, mixed cellularity; MUM1, melanoma-associated antigen (mutated) 1; NS, nodular sclerosis; THCRLBCL, T-cell/histiocyte-rich large B-cell lymphoma.

The evolution of HL classification—from the morphological Rye system to the molecularly informed WHO 5th Edition and ICC—highlights the increasing role of integrated diagnostics in lymphoma pathology. These updates enhance diagnostic accuracy, clarify treatment pathways, and improve patient outcomes (Figure 2).

The 2022/2023 updates to the HL classification underscore the need for precision diagnostics in hematopathology. As classification systems continue to evolve, they provide a more accurate framework for research and personalized therapy.

## 4. Morphology of Classic Hodgkin Lymphoma

A defining feature of cHL is the presence of characteristic malignant cells known as HRS cells. These cells are typically large, frequently binucleated or multinucleated, with conspicuous eosinophilic nucleoli and abundant amphophilic to mildly basophilic cytoplasm. The well-known “owl’s eye” morphology—produced by two symmetrical nuclei with prominent nucleoli—serves as a key diagnostic marker for pathologists. Beyond this classic appearance, multiple morphological variants of HRS cells can occur, broadening the histopathological spectrum observed in cHL [16,45].

### 4.1. Morphological Features of HRS Cells

HRS cells are typically large, ranging from 15 to 45 μm in diameter, and exhibit significant cytological atypia. Their nuclei may appear bilobed or multinucleated, with distinct, inclusion-like nucleoli that stain brightly with hematoxylin and eosin (H&E). The cytoplasm is usually ample and lightly basophilic, giving the cells a pale appearance under the microscope [46,47,48]. Several variants exist: (1) mononuclear variants (Hodgkin cells) resemble HRS cells but have a single nucleus [47,49]; (2) mummified forms are characterized by smaller, shrunken, and apoptotic-appearing cells [47,49]; (3) lacunar cells, seen in the nodular sclerosis subtype, show clear cytoplasmic retraction artifacts that result in a lacuna or space around the nucleus [47,49]; anaplastic variants may resemble cells found in anaplastic large cell lymphoma (ALCL), complicating the differential diagnosis [47].

### 4.2. Subtype-Specific Morphological Features

Each histological subtype of cHL possesses characteristic morphological patterns that aid in diagnosis and classification.

NScHL is the most common subtype, marked by fibrous bands dividing lymphoid tissue into nodules [50,51]. Lacunar-type HRS cells predominate, and eosinophils, plasma cells, and neutrophils are common in the background [52].

MCcHL shows a diffuse infiltrate rich in classic HRS cells, eosinophils, histiocytes, plasma cells, and small lymphocytes. This subtype lacks the fibrous bands seen in NSCHL and is often associated withEBV infection [51,53].

LRcHL features scattered HRS cells in a background of small B lymphocytes. The HRS cells often display a classic morphology and are fewer in number, which can sometimes pose diagnostic challenges [53,54].

LDcHL is the rarest subtype, demonstrating numerous pleomorphic HRS cells with scant reactive infiltrate. This variant is more common in immunocompromised patients and has the worst prognosis among cHL subtypes [33,51].

**Figure 3:**
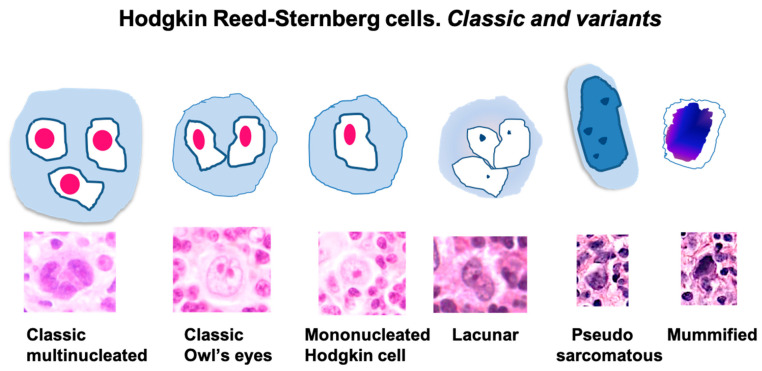
Hodgkin Reed-Sternberg cells (HRS). Classic and variants. HRS cells are large, multinucleated cells or binucleated “owl’s eye” cell, having huge eosinophilic nucleoli. In addition to these classic HRS cells there are several morphologic variants including the mononucleated, macronucleolated “Hodgkin cells”, the “lacunar cells”, that appear to be surrounded by a clear space or lacuna, the “pseudosarcomatous” cells and the “mummified” cells, that display condensed cytoplasm and pyknotic eosinophilic or basophilic nuclei. (reprinted from Carbone et al. [55]).

These differences are summarized in Table 2 and Figure 3, which provide a concise comparative overview of the morphological characteristics of each cHL subtype [55].

Table 2 outlines the various cytological variants of HRS cells observed in cHL. Each form is characterized by unique nuclear and cytoplasmic features that assist in subtype identification and differential diagnosis.

## 5. Clinical Relevance of Classification

The classification of HL carries substantial clinical importance, influencing diagnostic precision, therapeutic decision-making, and prognostic assessment. Far beyond academic interest, the current system supports real-time clinical management and ensures that treatment strategies are aligned with the biological characteristics of the disease.

### 5.1. Relevance for Diagnosis and Treatment Planning

Patients with NScHL often present with localized mediastinal involvement and typically respond well to standard frontline chemoradiotherapy, achieving excellent outcomes [56]. In contrast, LDcHL generally manifests with advanced-stage disease and an unfavorable prognosis, often necessitating more aggressive treatment regimens [57].

Similarly, LRcHL may closely resemble NLPHL both morphologically and immunophenotypically, making thorough immunohistochemical profiling critical to avoid misdiagnosis, as management approaches differ considerably [51]. While cHL usually responds well to ABVD-like chemotherapy protocols, NLPHL may be managed more conservatively or treated using regimens typically reserved for indolent B-cell lymphomas [35,58].

### 5.2. Prognostic Implications of EBV Positivity

Detection of EBV within HRS cells adds another dimension to cHL assessment. EBV-positive disease is more common in older patients, immunocompromised individuals, and populations in low-resource settings. Subtypes such as MCcHL and LDcHL are more often EBV-associated, which may correlate with poorer outcomes and underlying immune dysregulation. While the therapeutic application of EBV status remains investigational, its presence may inform future strategies incorporating immunomodulatory or antiviral therapies [59].

### 5.3. Role of CD30 and CD15 in Diagnosis and Therapy

From a diagnostic standpoint, CD30 and CD15 remain central markers for cHL classification. CD30 is uniformly expressed by HRS cells and represents the defining immunophenotypic hallmark. while a significant proportion also express CD15. PAX5 expression is typically weak, reflecting partial B-cell program retention, and CD20 expression may be seen in a subset of cases. The B-cell transcription factors OCT2 and BOB1 are particularly useful in challenging settings, as their expression is typically weak or absent in most cHL subtypes but is retained in LRcHL and strongly expressed in NLPHL, making these markers valuable in distinguishing these entities from other forms of cHL, which retain stronger B-cell phenotypes [7,53,60,61]. This immunophenotypic profile not only supports diagnosis but also provides a therapeutic target, most notably for brentuximab vedotin, an anti-CD30 antibody–drug conjugate that has reshaped both frontline and salvage treatment paradigms [62,63].

Although CD15 expression can vary, its detection helps differentiate cHL from other CD30-positive lymphomas such as ALCL or EBV-positive DLBCL, which typically lack CD15. Loss of CD30/CD15 expression or aberrant expression of markers such as CD20 or EMA may prompt diagnostic reconsideration, particularly in gray zone or composite lymphomas [64,65,66,67].

### 5.4. Combining Morphological and Molecular Approaches

While histopathological evaluation remains the foundation of cHL diagnosis, molecular pathology increasingly complements morphology, especially in diagnostically challenging cases. Techniques such as gene expression profiling, immunoglobulin gene rearrangement studies, and in situ hybridization for EBV-encoded RNA (EBER) can clarify ambiguous presentations, establish clonality, and uncover immune evasion pathways in EBV-positive disease [68,69].

With expanding access to advanced biomarkers and next-generation sequencing, the integration of genetic and transcriptional data is anticipated to further refine cHL classification, enabling more accurate risk stratification and the implementation of truly personalized therapeutic regimens.

### 5.5. Clinical Management

For many years, the standard of care for newly diagnosed cHL consisted of ABVD or BEACOPP chemotherapy with or without radiotherapy, with PET-adapted strategies refining intensity while maintaining excellent long-term remission rates [70]. Patients not cured by initial therapy were historically managed with salvage chemotherapy and autologous stem cell transplantation (ASCT), which offered durable remission in a subset [70]. In recent years, however, the treatment paradigm has shifted with the incorporation of novel agents. The phase III ECHELON-1 trial demonstrated that brentuximab vedotin plus AVD (BV-AVD) improved progression-free and overall survival compared with ABVD in advanced-stage disease, and BV-AVD has since become a standard of care in the frontline setting [71,72,73]. Most recently, the randomized S1826 trial established nivolumab-AVD (N-AVD) as a new frontline standard, with superior progression-free survival and tolerability compared with BV-AVD [74]. In the relapsed/refractory setting, PD-1 blockade has become firmly established: pivotal trials demonstrated high response rates and durable remissions with nivolumab and pembrolizumab, and the phase III KEYNOTE-204 trial showed pembrolizumab superiority over [70,75]. The prognostic impact of EBV in cHL is not uniform. Meta-analyses and cohort studies show but a neutral or modestly favorable effect in children and young adults, while EBV-positive cHL in immunodeficient patients represents a distinct poor-risk subset [76,77,78].

Thus, while traditional chemotherapy and ASCT remain curative backbones, the integration of BV and PD-1 inhibitors has redefined both frontline and salvage strategies, offering new standards of care in multiple clinical settings.

## 6. Tumor Microenvironment and Cellular Composition

Histopathological studies show that the TME in cHL plays a central role in disease biology. Although malignant HRS cells are scarce, they are embedded within a complex and reactive background of immune and stromal cells. This distinctive architecture highlights the dependence of HRS cells on their microenvironment for growth and survival, and underscores the TME as an active driver of disease progression rather than a passive scaffold [14,79].

HL presents a distinctive histological paradox: the malignant HRS cells usually account for less than 1–10% of the tumor mass, with the majority composed of reactive immune and stromal cells. This unique cellular arrangement underscores the critical role of the TME in disease development and progression. In cHL, the TME functions as an active, interactive network that supports tumor growth and influences the clinical and biological profile of the disease [14].

The composition of the TME differs notably between cHL subtypes—NScHL, MCcHL, LRcHL, and LDcHL—each displaying distinct histological and immunological characteristics.

In NSCHL, fibroblast-like stromal cells generate dense fibrous septa that divide the lymphoid tissue into nodules, creating the classic fibrotic architecture observed under the microscope [80]. MCcHL, on the other hand, contains a heterogeneous mixture of immune cells—eosinophils, plasma cells, neutrophils, mast cells, and both B and T lymphocytes—surrounding HRS cells, recruited through cytokines and chemokines secreted by the malignant population [16,81,82].

The rare LDcHL variant shows a paucity of small lymphocytes but an abundance of histiocytes and atypical HRS cells, along with irregular fibrosis and a less organized stromal framework compared to NScHL. LRcHL and NLPHL share a lymphocyte-rich TME, though NLPHL more frequently displays follicular dendritic cells and well-formed nodular structures [16,79].

**Figure 4:**
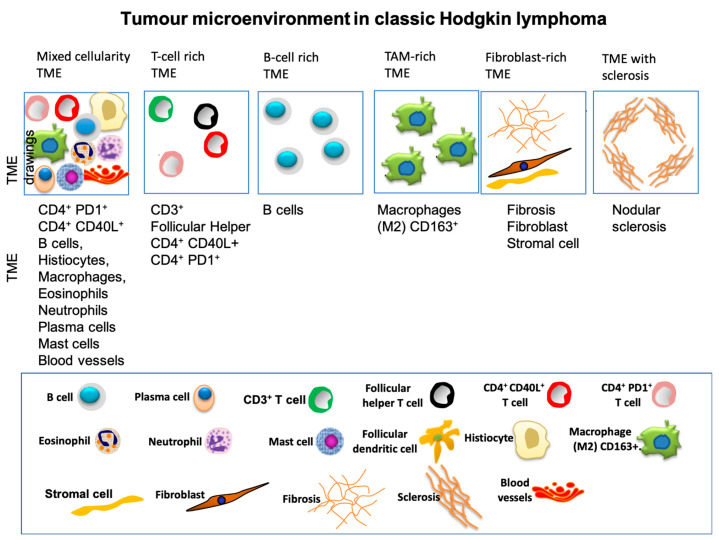
Tumour microenvironment (TME) in classic Hodgkin lymphoma (cHL). Drawings (top row) show cellular characteristics of different TME in cHL (reprinted from Carbone et al. [55]).

Although the TME in cHL displays a variable cellularity according to cHL subtypes, the TME frequently consists of a mixed, reactive infiltrate with B cells and T cells, neutrophils, histiocytes, plasma cells and mast cells. However, the composition of the TME can also be rich in T cells or B cells, or tumour associated macrophages (TAM) or fibroblasts associated with or not with band of sclerosis [55] (Figure 4).

The dynamic interplay between HRS cells and surrounding immune effectors further complicates the immune composition of the TME. HRS cells actively recruit and reprogram immune cells through the secretion of factors such as IL-5, IL-13, TARC, and others [83,84,85]. These molecules attract a range of cells, including T-helper 2 (Th2) cells, regulatory T cells (Tregs), eosinophils, and macrophages [86,87,88,89]. This recruitment not only sustains the inflammatory milieu but also contributes to immune evasion, as these cells can suppress cytotoxic immune responses [87].

HRS cells exhibit near-universal copy gains of chromosome 9p24.1/CD274 (PD-L1)/PDCD1LG2 (PD-L2), leading to copy number-dependent overexpression of these ligands and thereby establishing a strong genetic basis for enhanced PD-1 signaling [90,91]. Importantly, in addition to this genetically driven PD-L1 upregulation, HRS cells frequently lose MHC class I but retain MHC class II expression, allowing ongoing interactions with CD4^+^ T cells [92]. Within intact lymph node tissue, HRS cells are typically surrounded by PD-1^+^CD4^+^ T cells and PD-L1^+^ macrophages, forming a spatially organized immunoprotective niche that supports immune evasion and responsiveness to PD-1 blockade [93,94].

Macrophages, particularly TAMs, are prominent in many cHL cases [95,96]. Their presence has been linked to worse clinical outcomes, and they often express PD-L1, further aiding immune suppression within the TME. These PD-L1–positive macrophages are typically found in close proximity to HRS cells, enabling direct cell-cell interactions that may shield the tumor from immune attack [93,96,97].

Recent immunophenotypic and molecular studies have shed light on the presence of Th1-skewed CD4+ T cells and exhausted PD1+ T cells within the TME [94,98]. These findings revise the long-held notion that Th2 cells dominate in cHL [94]. Notably, Th1-polarized regulatory T cells and CD4+PD1+ T cells appear to play a central role in creating an immunosuppressive environment that benefits tumor survival [99].

The crosstalk between HRS cells and their microenvironment involves a multitude of ligand-receptor interactions. HRS cells express receptors such as CD30, CD40, and PD-L1, while their ligands—CD30L, CD40L, and PD1—are displayed by surrounding inflammatory cells, including eosinophils, mast cells, and T cells. This interactive network sustains HRS cell survival and fosters immune evasion mechanisms.

In cHL, immune escape is driven by recurrent genetic and viral mechanisms that also contextualize the success of PD-1 blockade. Focal/arm-level gains at 9p24.1 co-amplify PD-L1/PD-L2 with JAK2, resulting in ligand overexpression and JAK-STAT pathway reinforcement [14]. Loss-of-function alterations in B2M override MHC class I and impair CD8^+^ T-cell recognition, whereas structural variants and rearrangements involving CIITA reduce MHC class II expression and limit CD4^+^ T-cell help [100,101,102,103]. Constitutive activation of NF-κB (e.g., via TNFAIP3/A20 inactivation or REL dysregulation) and JAK-STAT signaling (e.g., JAK2/STAT6 alterations) sustains HRS-cell survival and the immunosuppressive milieu [14,101]. In EBV-positive cHL, LMP1 mimics CD40 signaling to activate NF-κB and induce PD-L1, while LMP2A provides surrogate B-cell receptor signals that promote survival of crippled germinal-center B-cell progenitors [101,104]. Together, these lesions explain the characteristic TME-centric biology of cHL and provide the molecular rationale for checkpoint inhibition.

## 7. Unresolved Issues

Table 3 summarizes the key unresolved questions in HL.

### 7.1. Unresolved Issues in the Classification of Hodgkin Lymphoma

Despite well-established WHO criteria distinguishing cHL from NLPHL, certain borderline cases display overlapping morphological and immunophenotypic features, making precise classification challenging. Current systems rely heavily on histology and immunohistochemistry, with limited integration of genomic and epigenomic data, which could provide deeper resolution. Furthermore, significant intratumoral heterogeneity within a given subtype is not adequately addressed by existing frameworks. In addition, mediastinal NScHL, mediastinal grey zone lymphoma (MGZL), and primary mediastinal B-cell lymphoma (PMBL) likely represent a biological and genetic continuum with overlapping features. Their distinction relies on architecture, inflammatory background, and degree of B-cell program expression, though diagnostic boundaries remain challenging in practice. A diffuse growth pattern does not exclude cHL if cytologic and immunophenotypic features are consistent, whereas centroblastic morphology with loss of cHL architecture favors MGZL. Assessment of multiple B-cell markers, rather than CD20 alone, is essential. The classification of cHL-like cases with strong, uniform B-cell marker expression as MGZL remains debated and requires further study.

The role of EBV status, although prognostically relevant, remains insufficiently incorporated into formal classification schemes.

### 7.2. Unresolved Aspects of HRS Cell Morphology and Phenotype

While HRS cells are generally accepted to originate from germinal center B cells, the exact molecular cascade leading to the near-complete loss of the B-cell transcriptional program remains incompletely understood. Phenotypic variability in the expression of markers such as CD30, CD15, PAX5, MHC class I, and MHC class II is observed both across patients and within individual tumors. Predictive biomarkers capable of reliably identifying patients who will benefit from targeted agents—such as PD-1 inhibitors or anti-CD30 therapies—are lacking. Moreover, the causal relationship between phenotypic diversity and TME modulation remains to be fully elucidated.

### 7.3. Unresolved Therapeutic Implications of TME Variability

A defining feature of cHL pathogenesis is the capacity of HRS cells to reshape immune interactions to favor tumor persistence. Instead of being eradicated by host immunity, they secrete cytokines and chemokines that selectively recruit regulatory T cells, macrophages, and other immunosuppressive cell types. These infiltrating populations, in turn, deliver trophic signals and shield HRS cells from cytotoxic attack. This reciprocal signaling loop enables the malignant clone to thrive within an otherwise immunologically hostile setting.

Although it is well established that TME composition affects prognosis and therapeutic response in cHL, no validated predictive model currently exists to guide treatment decisions based on specific TME profiles. The relative contribution of individual cellular populations—such as M2-polarized macrophages, regulatory T cells, and cancer-associated fibroblasts—to therapeutic resistance or sensitivity is not fully defined. The TME is also highly dynamic, undergoing shifts during disease progression or after therapy, complicating efforts to identify stable therapeutic targets. Strategies aimed at reprogramming or depleting specific pro-tumorigenic components remain experimental, with long-term efficacy and safety yet to be determined.

## 8. Conclusions

The classification of HL has evolved remarkably, from early morphology-based schemes such as Rye, through the biologically informed REAL and successive WHO editions, to the most recent WHO-HAEM5 and ICC frameworks. Each step has reflected a deeper understanding of the disease and progressively refined the separation of cHL from NLPHL. These refinements are not only of historical interest but continue to shape modern diagnostic practice.

cHL exhibits a distinct biological and pathological profile within hematologic cancers. Its uniqueness lies not only in the specific characteristics of the malignant HRS cells but also in the striking predominance of non-malignant cells within the tumor mass. In most cases, HRS cells account for less than 1–10% of the total tumor population, while the majority consists of a dense inflammatory and immune infiltrate collectively termed TME. Far from being a passive scaffold, the TME actively contributes to tumor growth, immune escape, and resistance to therapy.

The central role of the TME in cHL is highlighted by the dependence of HRS cells on surrounding immune and stromal elements for both survival and proliferation. These malignant cells display a profoundly altered B-cell transcriptional program and an atypical immunophenotype, marked by strong expression of CD30 and CD15 while frequently lacking canonical B-cell markers such as CD20. Such aberrant profiles often arise from genetic and epigenetic disruptions that block normal B-cell differentiation. These include suppression of transcription factors like OCT2 and BOB1—key regulators of B-cell identity—and constitutive activation of major signaling cascades, including NF-κB and JAK/STAT. Together, these alterations underpin the transformed state of HRS cells and their ability to manipulate their microenvironment.

Histopathological studies demonstrate that the composition and organization of the TME vary considerably across cHL subtypes. For instance, the MC subtype is characterized by a heterogeneous infiltrate of B and T lymphocytes, neutrophils, eosinophils, and macrophages, whereas the NS variant displays extensive fibrosis, fibroblast-like stromal populations, and broad collagen bands that compartmentalize tumor areas. These morphologic differences are not merely diagnostic markers—they also have potential implications for therapeutic responsiveness and prognosis.

## Figures and Tables

**Table 1:** Principal classification differences between WHO-HAEM5 and the ICC in the Hodgkin lymphoma area.

Topic	WHO 5th Edition (WHO-HAEM5)	ICC (2022)
**Top-level placement**	“Hodgkin lymphoma” family includes cHL and NLPHL.	“Hodgkin lymphoma” includes cHL; NLPHL is moved/renamed to NLPBCL under B-cell lymphomas.
**cHL subtypes**	Four subtypes retained: NScHL, MCcHL, LRcHL, LDcHL.	Same four subtypes retained.
**LRcHL**	LRcHL is a true cHL subtype; may mimic NLPHL morphologically but is not an intermediate or hybrid entity.	Same.
**NLPHL/NLPBCL**	Kept within HL as NLPHL (distinct entity).	Reclassified as NLPBCL (mature B-cell lymphoma).
**Relationship to THRLBCL**	Notes overlap/transition; encourages recognizing THRLBCL-like (diffuse) areas when present.	Emphasizes continuum with THRLBCL; both NLPBCL and THRLBCL are in large B-cell lymphoma group.
**Gray-zone interface**	MGZL placed among large B-cell lymphomas at the PMBL–cHL interface.	Same.
**Reporting emphasis**	Strong on integrated diagnosis (morphology + IHC ± genetics); retains historical continuity for trials.	Adds granular pattern reporting (e.g., NLPHL/NLPBCL variant patterns, diffuse components) to inform prognosis/management.
**Nomenclature & usage**	Uses NLPHL; cHL subtype names as above.	Uses NLPBCL; same cHL subtype names.

Abbreviations. cHL, classic Hodgkin lymphoma; LDcHL, lymphocyte depleted classic Hodgkin lymphoma; LRcHL, lymphocyte-rich classic Hodgkin lymphoma; MCcHL, mixed cellularity classic Hodgkin lymphoma; MGZL, mediastinal gray-zone lymphoma; NLPBCL, nodular lymphocyte predominat B-cell lymphoma; NLPHL, nodular lymphocyte predominat Hodgkin lymphoma; NScHL, nodular sclerosis classic Hodgkin lymphoma; PMBL, primary mediastinal B-cell lymphoma; THRLBCL, T/histiocyte-rich large B-cell lymphoma.

**Table 2:** Lymphocyte predominant (LP) cells and distinct morphological variants of Hodgkin Reed–Sternberg (HRS) cells.

Cells	Distinctive Morphological Features	Commonly Associated Subtype(s)
**LP (Popcorn) cell**	Multilobated nucleus with small, distinct nucleoli; B-cell immunophenotype	NLPHL
**Hodgkin cell**	Large mononuclear cell with prominent nucleolus	All cHL subtypes
**Lacunar cell**	Cytoplasmic retraction with clear perinuclear space (“lacuna”), often due to fixation artifact	NSCHL
**Mummified cell**	Shrunken cell body with condensed, hyperchromatic (pyknotic) nuclei	Advanced stages of cHL
**Pleomorphic HRS cell**	Markedly irregular nuclear contours with variable nucleolar size	MCcHL, advanced disease

Abbreviations. cHL, classic Hodgkin lymphoma; HRS, Hodgkin Reed-Sternberg; LP, lymphocyte predominant; MCcHL, mixed cellularity classic Hodgkin lymphoma; NScHL, Nodular Sclerosis Hodgkin Lymphoma; NLPHL, nodular lymphocyte-predominant Hodgkin lymphoma.

**Table 3:** Key unresolved questions in Hodgkin lymphoma research.

Area	Unresolved Issues	Details
**1. Classification of Hodgkin Lymphoma**	Lack of universally accepted subtyping criteria	Evolving definitions blur boundaries between subtypes.
	Clinical significance of rare or borderline variants	Intermediate features between cHL and NLPHL complicate diagnosis and treatment
**2. Morphological and Phenotypic Specificity of HRS Cells**	Mechanisms underlying phenotype heterogeneity	Inconsistent antigen expression (CD15/CD30; occasional CD20) not fully explained by genetics/epigenetics
	Role of disrupted B-cell program	Loss of B-cell identity markers (OCT2, BOB1) may affect prognosis and immune evasion, remains unclear
**3. Therapeutic Relevance of TME Variability**	Impact of cellular composition on treatment response	Tregs, macrophages, eosinophils variably influence chemotherapy, immunotherapy, and targeted agent outcomes
	Predictive value of TME-based stratification	Potential role of TME profiling in patient risk stratification and therapy selection remains under study

Abbreviations. HRS, Hodgkin Reed-Sternberg; TME, tumor microenvironment
